# Continuous Exposure to a Novel Stressor Based on Water Aversion Induces Abnormal Circadian Locomotor Rhythms and Sleep-Wake Cycles in Mice

**DOI:** 10.1371/journal.pone.0055452

**Published:** 2013-01-30

**Authors:** Koyomi Miyazaki, Nanako Itoh, Sumika Ohyama, Koji Kadota, Katsutaka Oishi

**Affiliations:** 1 Biological Clock Research Group, Biomedical Research Institute, National Institute of Advanced Industrial Science and Technology (AIST), Tsukuba, Japan; 2 Agricultural Bioinformatics Research Unit, Graduate School of Agricultural and Life Sciences, The University of Tokyo, Tokyo, Japan; Vanderbilt University, United States of America

## Abstract

Psychological stressors prominently affect diurnal rhythms, including locomotor activity, sleep, blood pressure, and body temperature, in humans. Here, we found that a novel continuous stress imposed by the perpetual avoidance of water on a wheel (PAWW) affected several physiological diurnal rhythms in mice. One week of PAWW stress decayed robust circadian locomotor rhythmicity, while locomotor activity was evident even during the light period when the mice are normally asleep. Daytime activity was significantly upregulated, whereas nighttime activity was downregulated, resulting in a low amplitude of activity. Total daily activity gradually decreased with increasing exposure to PAWW stress. The mice could be exposed to PAWW stress for over 3 weeks without adaptation. Furthermore, continuous PAWW stress enhanced food intake, but decreased body weight and plasma leptin levels, indicating that sleep loss and PAWW stress altered the energy balance in these mice. The diurnal rhythm of corticosterone levels was not severely affected. The body temperature rhythm was diurnal in the stressed mice, but significantly dysregulated during the dark period. Plasma catecholamines were elevated in the stressed mice. Continuous PAWW stress reduced the duration of daytime sleep, especially during the first half of the light period, and increased nighttime sleepiness. Continuous PAWW stress also simultaneously obscured sleep/wake and locomotor activity rhythms compared with control mice. These sleep architecture phenotypes under stress are similar to those of patients with insomnia. The stressed mice could be entrained to the light/dark cycle, and when they were transferred to constant darkness, they exhibited a free-running circadian rhythm with a timing of activity onset predicted by the phase of their entrained rhythms. Circadian gene expression in the liver and muscle was unaltered, indicating that the peripheral clocks in these tissues remained intact.

## Introduction

Exposure to stress is related to an increase in the incidence of various psychiatric illnesses, anxiety, and mood disorders in humans [1]. Mood disorders are closely associated with circadian rhythm abnormalities and sleep disorders [2]. Circadian genetic sleep disorders, including familial advanced sleep phase syndrome and delayed sleep phase syndrome, are often comorbid with depression [3]. Collectively, abnormal circadian rhythms, sleep disorders, and psychiatric illnesses can be inseparably linked and mutually regulated. The circadian clock regulates the daily rhythms of the sleep/wake cycles, body temperature (BT), hormone levels, and even cognition, attention, and mood. Experimental ablation of the suprachiasmatic nucleus (SCN) of the anterior hypothalamus abolishes the circadian rhythmicity of locomotor activity, sleep-wake cycles, and BT in mice and rats [4]–[6]. The molecular basis of circadian rhythm generation has been extensively studied, and circadian rhythms are generated from interacting transcriptional translational feedback loops involving clock genes such as *Clock*, *Bmal1*, *Periods*, and *Crys* [7]. Mood stabilizers, such as lithium chloride, which are used to treat these disorders, can also restore the circadian clock [8]. Lithium chloride inhibits GSK3β activity and stabilizes PER2 protein [9], whereas chronic stress reduces PER2 protein expression in the brains of mice with reduced activity [10], [11].

Mouse models of sleep disorders have historically been based on sleep deprivation. Chronic sleep deprivation, such as via the classic disk-over-water technique and slowly rotating wheels, cannot be maintained for periods of 48 h or repeated for 20 h/day for 5 days because survivability decreases [12], [13]. Furthermore, sleep deprivation stress forces mice to remain awake. Temporal exposure to conventional stressors, such as immobilization, electric shock, gentle handling and food deprivation, at fixed times of the day affects sleep time and consolidation [14], while the timing of stress exposure provides mice with zeitgeber cues that induce stress anticipation activity [15] and can affect circadian rhythmicity. Chronic mild stress (CMS), a combination of conventional mild stress and repeated social defeat stress applied temporally to animals once daily, promotes depression-like behavior and reduces total locomotor activity and the amplitude of such activity. However, none of these sleep-deprivation or conventional stressors is appropriate for studying circadian locomotor rhythms. Animal models for studying the stress-induced dysregulation of circadian locomotor rhythms have not been established because most conventional methods of inducing stress cannot be implemented while recording locomotor activity.

To clarify these issues, we developed a novel method to impose continuous mild stress on mice and examined its impact on entrained and free-running activity/rest cycles, BT rhythms, sleep architecture, and molecular circadian clock systems. Activity/rest cycles were profoundly disrupted, total locomotor activity was reduced, and activity became redistributed from the nighttime to early daytime. The nocturnal maintenance of a higher core BT was attenuated in the stressed mice. Sleep architecture was also damaged insofar as the sleep/wake rhythms became blunted and sleep duration was fragmented. However, stress did not affect either free-running periodicity under constant darkness or the molecular oscillation of clock genes, including *Per2* and *Bmal1*, in peripheral tissues.

## Materials and Methods

### Animals

Male C3H-HeN mice purchased from Japan SLC Inc. (Hamamatsu, Japan) were housed under a 12-h light: 12-h dark cycle (lights on at 08:00) at a controlled ambient temperature of 24±1°C and provided with food (CE-2, CLEA Japan Inc., Tokyo, Japan) and water *ad libitum* throughout the study. Food intake during the study was determined by weighing the remains of a known daily excess of food at Zeitgeber time (ZT) 3. Chow spilled into the cages was not recovered under the conditions of perpetual avoidance of water on a wheel (PAWW) stress. New chow was exchanged for leftover food and we confirmed the absence of chow spilled in the cages every day to minimize the loss of spilled chow throughout the study. When we found a block of chow in the cage, we omitted the data from scoring. The mice were also weighed weekly when we measured the chow. Food intake is expressed as the average daily intake for 5 days per week (n = 6). The experiments were conducted in accordance with the guidelines for the Care and Use of Laboratory Animals at the National Institute of Advanced Industrial Science and Technology (AIST), and the Animal Care and Use Committee at AIST approved the study protocol (approval no. 2011 - 056b).

### Behavior activity monitoring and continuous mild stress

**Figure 1:**
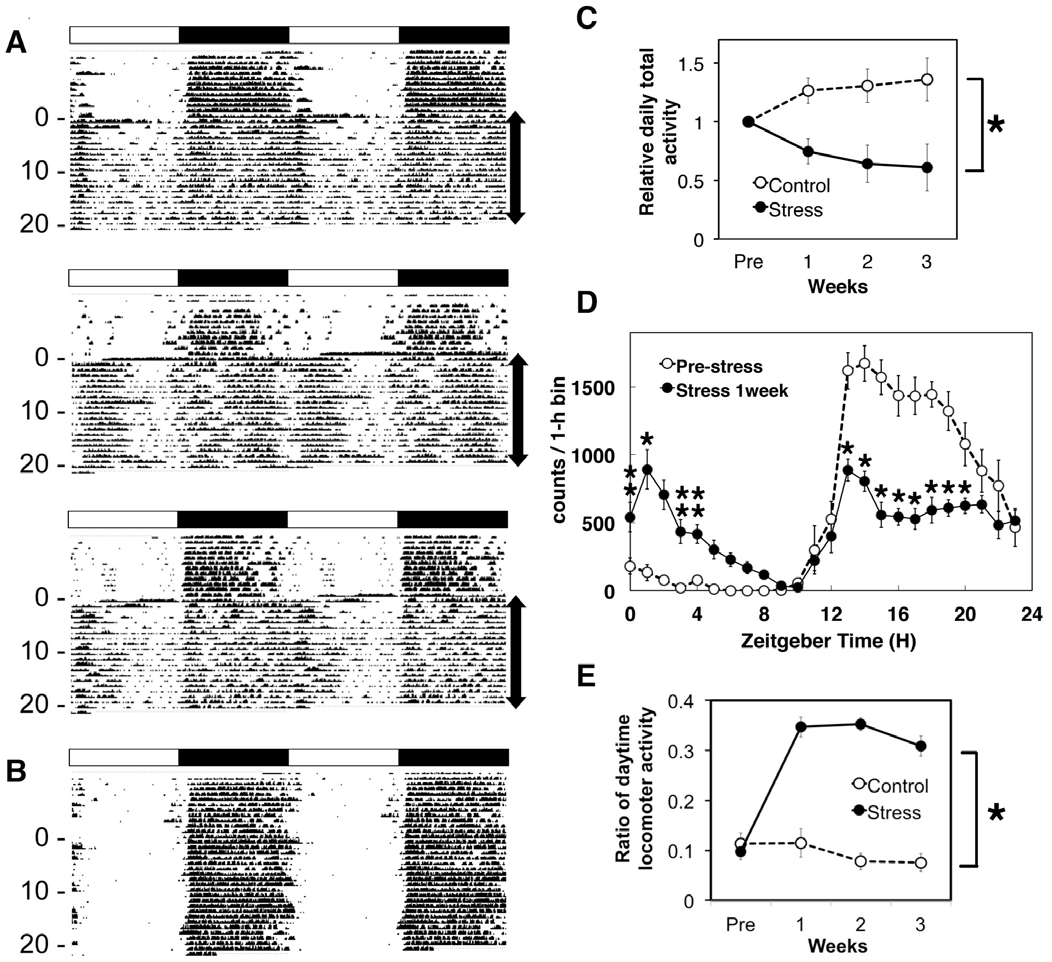
PAWW stress desrupted circadian locomotor activities. Representative double-plot actograms for mice under continuous mild stress (A) and control mice (B) under L:D. The light/dark cycles are shown as white/black bars, respectively, on each actogram. The numbers on the left indicate the days of exposure to PAWW stress and the arrows on the right indicate periods of PAWW stress. The circadian rhythms of locomotor activity in pre-stress mice and in control mice were well organized and became obscure after they were transferred to the cages in which PAWW stress was imposed. (C) Daily total locomotor activity was gradually, but time-dependently, reduced in the mice exposed to PAWW stress (closed circles with a solid line) compared with the non-stressed mice (open circles with a dashed line). Daily total activity was averaged for 7 days within the pre-stress period and after 1, 2, and 3 weeks of PAWW stress. Relative daily total activity is expressed as relative to the averaged activity of the pre-stress period. Values are shown as means ± SEM (n = 12). RM two-way ANOVA revealed a significant main effect of stress on locomotor activity (*P<0.01). (D) Wheel running activity in mice before (pre-stress, open circles) and during 1 week of PAWW stress (closed circles) plotted over 24 h. Wheel activity in 1-h bins was averaged for 6 days before or from days 2–7 of exposure to PAWW stress, and the mean values from individual mice were collated to yield a single 24-h profile. Values are shown as means ± SEM (n = 6). Distribution of diurnal locomotor activity was changed by PAWW stress. RM two-way ANOVA revealed no significant main effect of stress on locomotor activity, while the post hoc Tukey's test indicated significant differences (*P<0.05, **P<0.01). (E) Daytime activity relative to total locomotor activity. Open and closed circles, counts from the non-stressed and continuously stressed mice, respectively. Approximately 10% of the total daily activity occurs during the daytime in the non-stressed mice. Values are shown as means ± SEM (n = 12). Daytime activity was enhanced by PAWW stress by up to 30% (*P<0.001, RM two-way ANOVA).

Mice (male, 8–15 weeks old) were maintained individually in plastic cages containing animal paper bedding and running wheels (SW-15; Melquest, Toyama, Japan) and they were provided with conventional food and water *ad libitum* for 1–2 weeks before exposure to stress and wheel activity recording. On the first day of exposure to stress, we exchanged the paper bedding with water to a depth of 1.5 cm at room temperature. The mice were averse to becoming wet and therefore chose to remain on the wheel almost all day and rarely entered the water under these conditions. The cages were placed under conventional light on ventilated shelves (6 cages on 1 shelf) and the water in the cages was exchanged 3 times weekly to keep the bedding clean. The mice accessed food from the wheels or directly from a stainless steel wire top feeder. Drinking water was accessible from the wheel. We named this model stressor PAWW. Activity was monitored even under exposure to the stressor. The lighting conditions were changed in some experiments from light/dark (L:D) to constant darkness (D:D). Wheel running activity in 1-min bins, which was monitored using a Chronobiology Kit (Stanford Software Systems, Santa Cruz, CA), is displayed as double-plot actograms, as described previously [16]. The free-running periods of individual mice were determined using a **χ^2^** periodogram. The wheel activity of each mouse was accumulated in 1-h bins for 7 days before, during, and after stress exposure, and then averaged to yield a single 24-h profile. Exposure to PAWW stress affected locomotor activity acutely on the first day, when all mice were active essentially throughout the entire day. Thus, we omitted the data collected on the first day of exposure to PAWW stress to create a 1-week stress profile (Figure 1D).

### BT measurements

Core BT was continuously measured every 5 min as described previously [17] using an intraperitoneal transmitter (TA10TA-F20; Data Science International, St. Paul, MN) implanted under isoflurane anesthesia. Data from 24 h on the day before and the 6^th^ day of PAWW stress were averaged over 1-h intervals. Deviations in BT from the average temperature at each time point (every 5 min for 1 h) were measured for 6 h and are scored as the instability index of BT.

### RNA extraction and quantitative real-time PCR

The liver and muscle were collected from each mouse, snap-frozen in liquid nitrogen, and stored at −80°C. Total RNA was extracted from the frozen tissues using RNAiso (Takara Bio Inc., Otsu, Japan) and cDNA was generated using PrimeScript RT Master Mix (Takara Bio Inc., Otsu, Japan). Quantitative real-time PCR proceeded using SYBR^®^ Premix Ex Taq™ II (Takara Bio Inc., Otsu, Japan) and a LightCycler™ (Roche Diagnostics, Mannheim, Germany). The PCR conditions comprised 10 s at 95°C followed by 45 cycles of 5 s at 95°C, 10 s at 58°C, and 10 s at 72°C. The endogenous quantity control for the liver was β-actin, and that for muscle cDNA was GAPDH, as described previously [18]. Each value was normalized to reference gene mRNA expression. The primer sequences were: *Per2*, 5′-GGCACATCTCGGGATCG-3′ and 5′-GAGCAGAGGTCCTCGCC-3′; *Bmal1*, 5′-GGAGAAGGTGGCCCAAA-3′ and 5′-AGGCGATGACCCTCTTA-3′; *β-actin*, 5′-CACACCTTCTACAATGAGCTGC-3′ and 5′-CATGATCTGGGTCATCTTTTCA-3′; and *gapdh*, 5′-GACCTCAACTACATGGTCTACA-3′ and 5′-ACTCCACGACATACTCAGCAC-3′.

### Tissue and blood collection and plasma analysis

The mice were sacrificed at 6- or 4-h intervals across the L:D cycle to obtain blood samples. Blood was collected by puncturing the inferior vena cava under inhalational anesthesia, and transferred into tubes coated with EDTA. Corticosterone was quantified in the first 100 µL of collected blood. Blood samples were separated by centrifugation at 4,500×*g* at 4°C for 10 min and the supernatant was stored at −80°C. The levels of plasma corticosterone (AssayPro, St. Charles, MO), melatonin, epinephrine, norepinephrine (IBL International, Hamburg, Germany), and leptin (Morinaga Institute of Biological Science Inc., Kanagawa, Japan) were determined by enzyme-linked immunosorbent assays according to the manufacturers' instructions.

### Sleep recording and analysis

Two electroencephalographic (EEG) electrodes were implanted into the skull and fixed with dental cement, and then 2 stainless steel wires were implanted in the neck muscles to collect electromyographic (EMG) signals. A telemetric device (TL11M2-F20-EET; Data Sciences International, St. Paul, MN) for sleep recording was then implanted subcutaneously into the backs of the mice according to the manufacturer's instructions under pentobarbital (50 mg/kg i.p.) anesthesia. After 10 days of recovery in plastic cages containing paper bedding and a wheel, polygraphic EEG, EMG, and subcutaneous BT were continuously recorded for 48 h during the pre-stress day and the first day of exposure to PAWW stress. We also collected EEG and EMG data for 24 h under L:D on the 7^th^ day of exposure to PAWW stress.

Cortical EEG and EMG signals were digitized at a sampling rate of 500 Hz and recorded using Dataquest A.R.T.^TM^ (Data Sciences International, St. Paul, MN). Polygraphic recordings were automatically scored offline in 10-s epochs into 3 stages of wake, rapid eye movement (REM) sleep, and non-rapid eye movement (NREM) sleep using SLEEPSIGN (Kissei Comtec, Nagano, Japan) according to the standard criteria described below. Wakefulness was characterized by low amplitude EEG signals with mixed frequency components and relatively high, often irregular, EMG activity. The amplitude of EEG activity during NREM sleep was high and dominated by 1–4-Hz slow-frequency waves, while EMG activity was low. Low-amplitude EEG signals dominated by 6–9-Hz theta waves and low EMG activity characterized REM sleep. Defined sleep-wake stages were examined visually and corrected if necessary. The EEG power during all NREM sleep epochs was calculated as the ratio of the slow wave or delta frequency range (1–4 Hz). To correct for interindividual differences in the strength of the EEG signals, the power values of all animals were normalized by expressing them relative to the average baseline power in the same frequency band.

### Statistical analysis

All values are expressed as means ± SEM. Statistical analysis of the differences between groups in daily locomotor activity, body weight, food intake, BT, and wake/sleep length values were evaluated by two-way repeated measures (RM) analysis of variance (ANOVA). Univariate two-way ANOVA was used to evaluate the effect of PAWW stress on plasma leptin, corticosterone, and melatonin levels. When a statistical interaction was observed between factors, a comparison of all groups was performed using Tukey's post hoc test. Plasma corticosterone levels at ZT2 and catecholamine levels were evaluated using Student's or Welch's t-test. The effects of PAWW stress upon the circadian rhythmicity of plasma corticosterone levels and the expression of clock genes were analyzed by a cosinor method as described previously [19]. Comparisons of acrophase and amplitude were performed by Student's or Welch's t-test. The ratio of wake/REM sleep/NREM sleep duration between pre- and post-PAWW stress was evaluated using the G-square test. Statistical significance was established at P<0.05.

## Results

### Locomotor activity

Locomotor activity was monitored as wheel running in cages containing paper bedding during a 12-h light/12-h dark cycle (L:D). The mice were more active under L:D during the night and less active during the day, which is normal for nocturnal mice. On the first day of exposure to PAWW stress, the bedding in each cage was replaced with water to a depth of 1.5 cm. Thereafter, the mice remained on the wheel throughout the day and accessed food and water from the wheel to avoid contact with the water. Figure 1A shows that exposure to PAWW stress significantly changed wheel-running behavior. Total locomotor activity was significantly enhanced on the first day of exposure to PAWW stress, when the mice ran on the wheels almost all day. However, total daily locomotor activity declined within 2 days of PAWW stress, but the timing of both the onset and offset of activity was imprecise and the active period continued until 6 h after lights on (Figure 1A). Furthermore, the mice often remained active even during the latter half of the daytime, when the control mice scarcely moved (Figure 1B).

Total daily activity was reduced gradually in the mice exposed to PAWW stress over a period of 3 weeks, but gradually increased in the control mice (F(1,20) = 11.01, P = 0.003, Figure 1C). However, this reduction in locomotor activity was remarkable even during the first week of exposure to stress and it was not expressed equally across all times of the day and night (F(1,10) = 4.01, P = 0.073, Figure 1D). The distribution of activity was biphasic, with a burst of activity that was coincident with lights on and a second more profound and longer bout of activity just after lights off, when a general active phase was evident in the mice before exposure to PAWW stress. Both pre-stress and stressed mice exhibited consolidated locomotor activity that started at lights off; however, this activity began to diverge between the pre-stress and stressed mice at 3 h into the dark period at ZT15. The wheel running activity of the stressed mice began to decline to half-maximal where it remained for 10 h, whereas that in the pre-stress mice was sustained at a high level for the first 6 h and declined gradually during the last 6 h of darkness during the same period (Figure 1D). Exposure to PAWW stress obviously reduced the amplitude of behavioral activity over 24 h compared with control conditions. Accordingly, >30% and <10% of the total daily activity occurred during the light period in the PAWW-stressed and control mice, respectively (F(1,21) = 66.77, P<0.001, Figure 1E). These effects persisted for up to 3 weeks without adaptation. Locomotor activity was similarly abnormal in almost all mice tested, but varied intra- and inter-individually during the daytime in the PAWW-stressed mice (Figure 1A).

Locomotor activity recovered when the mice were transferred from stressful to normal cages. Figure S1A–C shows that 3 of the 4 tested mice did not fully recover locomotor activity in the dark phase during the first week after exposure to PAWW stress. However, the other mouse immediately regained a similar locomotor profile to that before exposure to PAWW stress, namely rest during the light period and locomotion during the dark period (Figure S1D).

### Food intake and body weight

**Figure 2:**
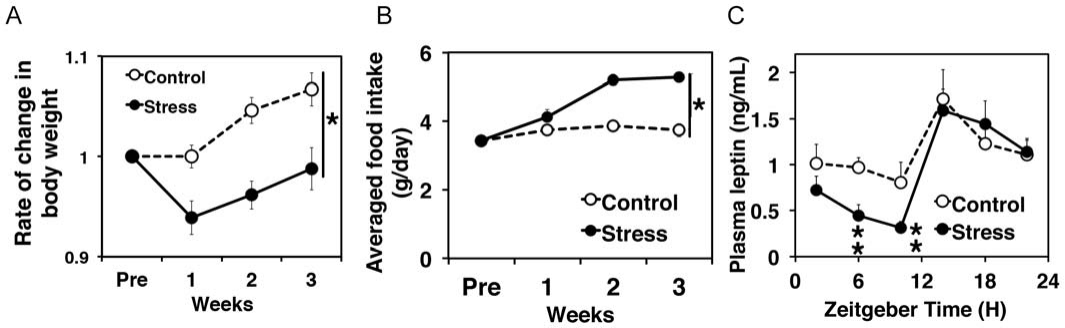
PAWW stress affected on body weight, and food intake. Effect of continuous PAWW stress on body weight (A), food intake (B), and levels of plasma leptin (C). All mice were weighed at ZT3. Values are shown as means ± SEM (n = 12 per group). The stressed mice weighed significantly less than the control mice after 1 week of exposure to PAWW stress (RM two-way ANOVA, *P<0.05) and remained significantly lighter over the next 3 weeks. The food intake of the stressed and control mice (mean ± SEM) differed significantly within 3 weeks (*P<0.01, RM two-way ANOVA). Diurnal plasma leptin levels in mice stressed for 1 week (continuous stress) and control mice (n = 6–8 at each time point). The black and white bars indicate light/dark conditions, respectively. Univariate two-way ANOVA revealed no significant main effect of stress on plasma leptin levels, while the levels were significantly lower during the light period in the stressed mice (**P<0.05, Student's t-test).

Chronic stress influences food intake and body weight [20]. The mice were weighed weekly from 1 week before until 3 weeks after starting exposure to PAWW stress. The average weekly rate of weight gain in the control mice was 2.6% (Figure 2A). In contrast, the mice lost a significant amount of weight during the first week of exposure to PAWW stress and then gained a little after the second week, but their weight gain was more moderate than in the control mice (F(1,21) = 7.01, P = 0.015, Figure 2A).

Food consumption increased notably within 2 weeks of PAWW stress and persisted over the time course of the experiment (F(1,21) = 21.98, P = 0.0001, Figure 2B). Accompanying this hyperphagia, daytime plasma leptin levels became significantly downregulated in the mice stressed for 1 week (Figure 2C, ZT6, P = 0.019 and ZT10, P = 0.046), while univariate two-way ANOVA test showed no significant difference between both groups (F(1,70) = 3.43, P = 0.068). Exposure to PAWW stress affected not only daily locomotor rhythmicity but also metabolic balance after 2 weeks. To minimize the metabolic and psychiatric effects of continuous stress on locomotor rhythmicity, we analyzed the homeostatic effects of abnormal locomotor rhythms caused by PAWW stress after 1 week, when the change in mouse locomotor activity was conspicuous.

### Blood levels of corticosterone, melatonin, epinephrine, and norepinephrine

**Figure 3:**
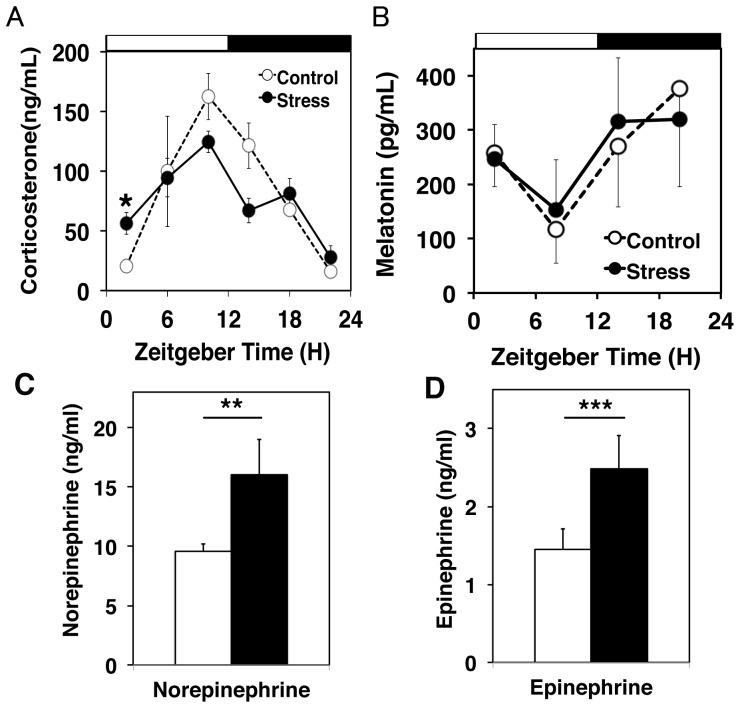
The effects of PAWW stress on plasma level of corticosterone, melatonin and catecholamines. Diurnal plasma corticosterone (A) and melatonin (B) levels (means ± SEM) in mice under continuous stress (closed circles, solid line) and controls (open circles, dashed line; n = 7 at ZT2 and 14, n = 3 at ZT8 and 20). The white/black bars indicate light/dark conditions, respectively. Univariate two-way ANOVA revealed that the effect of PAWW stress was not significant. Corticosterone levels were significantly increased at ZT2 and decreased at ZT14 (*P<0.05, Student's t-test). The plasma epinephrine (C) and norepinephrine (D) levels were determined in the stressed (solid bar) and control (open bar) mice at ZT2. Data are shown as means ± SEM (n = 6 per group). **P = 0.031, **P = 0.072 for averaged levels in the control mice vs. mice stressed for 7 days (Student's t-test).

The activity of the hypothalamic-pituitary-adrenal (HPA) axis is tightly controlled in a clock-dependent manner under normal conditions. The activity of the HPA axis is often acutely activated by exposure to conventional stress [9]. We measured plasma corticosterone levels in mice exposed to PAWW stress. Although corticosterone secretion increased acutely on the first day of PAWW stress (by 5.8- and 2-fold after 2 and 14 h of stress, respectively), the levels did not increase significantly after 1 week of continuous PAWW stress (F(1, 34) = 0.33, P = 0.56, Figure 3A). Cosinor analysis revealed that PAWW stress slightly reduced the amplitude of the corticosterone rhythm, but the levels and acrophase did not change significantly (Table S1). Corticosterone levels were significantly higher at ZT2 (P = 0.035).

The circadian rhythm of plasma melatonin is robust and closely associated with sleep homeostasis [21]. We found that continuous PAWW stress did not affect either plasma melatonin levels or its circadian rhythmicity (Figure 3B).

The enhanced locomotor activity in the daytime encourages speculation as to whether elevated sympathetic tone in the stressed animals contributes to sleep disturbance. We determined plasma epinephrine and norepinephrine levels because elevated sympathetic nerve activity often results in increased systemic levels of catecholamines. The levels of norepinephrine were significantly elevated (P = 0.031, Figure 3C), whereas those of epinephrine were only moderately and non-significantly increased in mice exposed to PAWW stress for 1 week (P = 0.072, Figure 3D).

### BT

**Figure 4:**
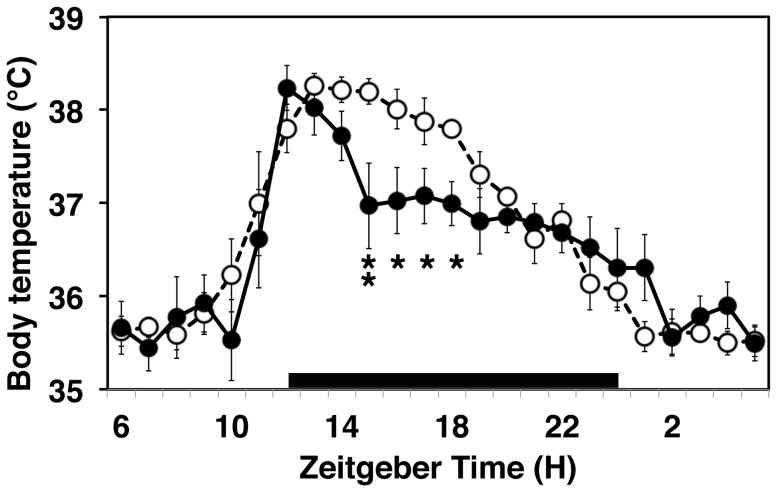
PAWW stress changed diurnal rhythms of core BT. Diurnal rhythms of core BT of mice on the day before (open circles, dotted line) and at 7 days of PAWW stress (closed circles, solid line). The core BT in 5-min bins was averaged hourly. Data are shown as means ± SEM (n = 6). Black bar, dark period of the day. After 1 week of PAWW stress, the diurnal rhythm of BT was similar to that during the pre-stress period, whereas maintenance of a higher BT during the dark period was disrupted. Averaged BT on day 7 of PAWW stress compared with that at 1 day before exposure to PAWW stress. RM two-way ANOVA indicated a significant interaction between time × stress (F(23,230) = 2.22, P = 0.002), and a significant effect of stress was observed during the nighttime (**P<0.01, *P<0.05, post hoc Tukey's analysis).

Exposure to PAWW stress induced blunted circadian locomotor rhythms, decreased body weight, and increased food consumption. These findings suggest that exposure to continuous PAWW stress was closely linked to a negative energy balance in mice, meaning that caloric intake was lower than caloric expenditure, which reflects thermoregulation. Therefore, we compared the rhythms of core BT in the mice between the day before and after 6 days of PAWW stress to determine whether or not circadian thermoregulation was altered. The shapes of the pre-stress and stressed BT rhythms were significantly different. Nocturnal elevation was organized and core BT was reduced during the daytime in pre-stressed mice. Although the BT of the stressed mice also became nocturnally elevated, it was not maintained throughout the nighttime (Figure 4). In addition, the decrease in BT during the latter half of the dark period and at the start of the light period in the pre-stressed mice, which might induce falling asleep, disappeared in the continuously stressed mice. The BT of each mouse plotted for 10 consecutive days changed remarkably at each time point during continuous PAWW stress compared with the pre-stressed state (Figure S2A). Furthermore, the deviation of each BT in 5-min bins from a corresponding average of 1 h was higher in the stressed mice compared to the control mice (F(1,10) = 21.88, P = 0.001, Figure S2B). These findings suggest that PAWW stress destabilizes thermoregulation or that it dysregulates locomotion, which in turn induces thermogeneration.

### Sleep/wake rhythms and EEG delta power

**Figure 5:**
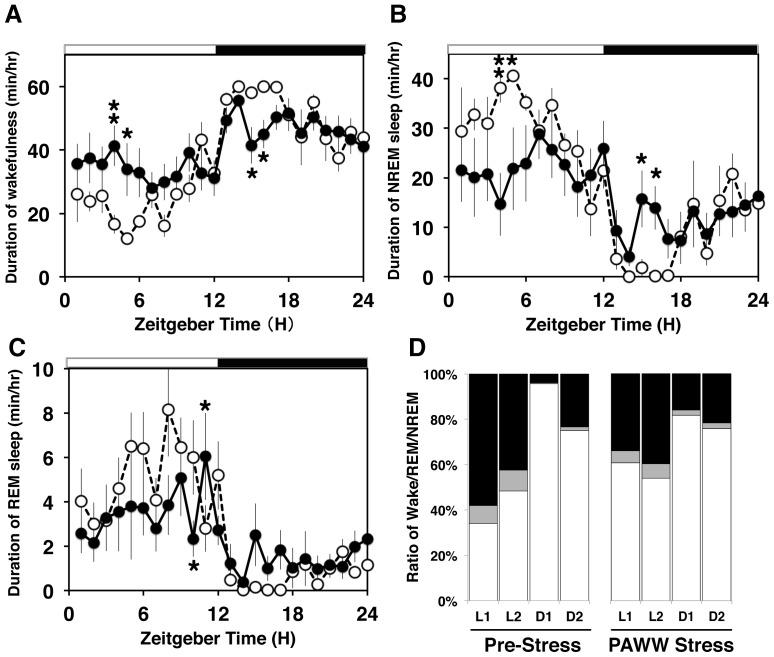
The effect of PAWW stress on diurnal rhythms of sleep. Hourly progression of time spent by mice in wakefulness (A) or in NREM (B) and REM (C) sleep over 24 h at 1 day before (open circles, dashed line) and on day 7 (closed circles, solid line) of PAWW stress (means ± SEM, n = 8). Black and white bars, light/dark conditions, respectively. Continuous stress reduced the duration of both REM and NREM sleep, especially during the first 6 h after lights on, and during wakefulness at 4 h after lights off. RM two-way ANOVA revealed a significant interaction between time × stress (wakefulness; F(23,230) = 2.22, P = 0.002, NREM; F(23,230) = 2.02, P = 0.005, REM; F(23, 230) = 1.93, P = 0.008). A significant simple main effect of stress treatment was observed (**P<0.01, *P<0.05). Wake/REM/NREM ratio at ZT0–5 (L1), ZT6–11 (L2), ZT12–17 (D1), and ZT18–23 (D2) under pre- and PAWW stress conditions (D). Sleep time distribution––daytime rest and nighttime activity––was rhythmic on the pre-stress days. Continuous stress caused wake epochs to appear frequently during the daytime and significantly increased the duration of NREM sleep during the nighttime, as compared with control mice. G-square test between pre- and post-stress showed significant changes (P<0.01) for the two phases of L1 and D1.

Circadian sleep-wake rhythms were precise under baseline conditions, with more sleep during the light period than during the dark period, as reported previously [22]. However, sleep architecture and sleep-wake rhythms were remarkably affected in the stressed mice in a similar manner to their circadian locomotor activity. Seven days of exposure to PAWW stress obviously affected the daily sleep-wake cycles (Figure 5). Continuous stress increased wakefulness for 6 h after lights on (when mouse activity is usually masked and they fall asleep), whereas NREM sleep was significantly reduced (Figure 5A, B, and D). These findings correspond to the difficulties of falling asleep associated with insomnia. Conversely, the duration of wakefulness and NREM sleep was reduced in mice under continuous PAWW stress during the first half of the nighttime, when normal mice are fully awake and highly active (Figure 5A, B, and D). These results suggest that continuous stress induced sleepiness in the mice during the active phase, which is the typical phenotype of insomnia. The duration of REM sleep in the stressed mice was reduced during the light period and increased during the dark period, although the difference did not reach significance (Figure 5C and D).

The sleep/wake rhythms differed between days 1 (acute phase) and 7 (chronic phase) of exposure to PAWW stress. Figure S3A shows that acute stress significantly reduced the total duration of NREM and REM sleep, which was accompanied by an increase in wakefulness (P<0.05). Continuous stress exerted a treatment effect upon sleep-wake time, but significant differences were scant (Figure S3A). The total duration of wakefulness, NREM sleep, and REM sleep did not completely recover to pre-stress levels.

We compared hypnograms between the mice before and during continuous PAWW stress to further confirm whether continuous stress affects sleep architecture. Figure S3B shows that the continuously stressed mice underwent far more state transitions during both the light and dark periods than the pre-stressed mice. Representative hypnograms show that NREM sleep was interrupted by numerous awakenings during the daytime, and that bouts of activity during the nighttime were often interrupted by short periods of NREM sleep. Thus, the frequency of sleep-wake transitions increased during the day and the daily sleep-wake cycles resembled those of wheel-running locomotor activity (Figure 1A). As shown in Figure S3, the averaged EEG delta power during NREM sleep was reduced under continuous stress in 6 of the 9 tested mice compared with the pre-stress period to 87.65±3.8% for 24 h (normalized by the baseline period), whereas the levels were similar to the pre-stress state in the remaining 3 mice (107±3.5%). Averaged EEG in NREM sleep during L1 (68.8±10.1%) and D2 (63.2±3.4%) was more severely disrupted than during L2 (93.7%±12.5%) and D1 (87.4±21.1%) in the stressed mice. These data suggested that continuous stress affects not only the duration of sleep but also its quality.

### Circadian clock system in mice under PAWW stress

**Figure 6:**
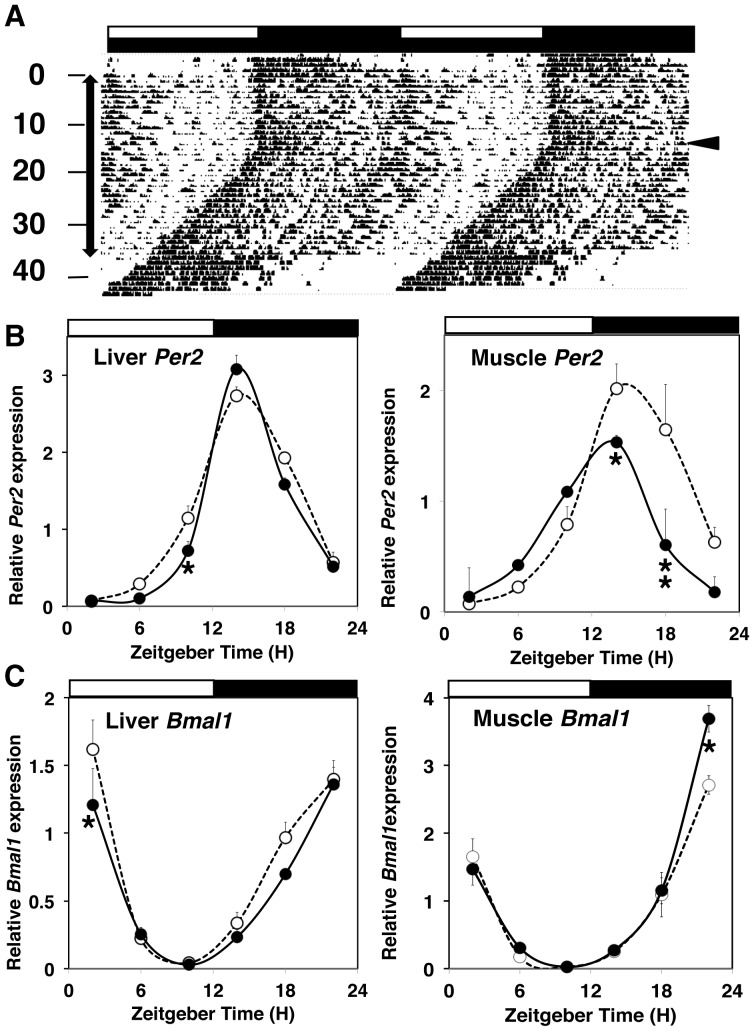
The effect of PAWW stress on circadian clock. Continuous stress similarly affects circadian rhythm behavior under constant darkness (D:D) and under light/dark (L:D) conditions (A). Light/dark cycles are shown as white/black bars on the actogram. The numbers and the arrow on the left indicate days of exposure to PAWW stress and periods of PAWW stress, respectively. The arrowhead on the right indicates the transition from L:D to D:D. Circadian oscillation of clock genes, *Per2* (B) and *Bmal1* (C) in the liver (left) and muscle (right) of the stressed (closed circles) and control mice (open circles). The black and white bars indicate light/dark conditions respectively. Univariate two-way ANOVA did not reveal a significant difference. The asterisks indicate a significant difference between the control and stress conditions by post hoc Tukey's analysis. Relative expression of each gene in the tissues at each time point in 4 mice (means ± SEM).

Rhythmic activity was entrained to the L:D cycle in mice even under continuous PAWW stress (Figures 1 and 6). When the mice were kept in D:D under continuous PAWW stress, endogenous circadian rhythmicity was sustained with a tau value of 23.66±0.04 h, and activity onset was predicted by the phase of their entrained rhythms (Figure 6A). These results suggested that PAWW stress did not affect the circadian clock system of the central pacemaker oscillator that resides in the SCN. Furthermore, the hyperactivation induced by the stressor at circadian time 1–4 was also evident even under D:D. This indicated that locomotor activation during the daytime in the stressed mice was not a result of their enhanced susceptibility to light. When the mice were released from PAWW stress under D:D, circadian rhythmicity was sustained with a tau value of 23.62±0.11 h, whereas locomotor activity completely disappeared during the subjective day (Figure 6A).

Corresponding to the integrity of the circadian locomotor rhythm, circadian oscillations in the gene expression of *Per2* and *Bmal1* in peripheral tissues, including liver and muscle, were robust in both the stressed and control mice under L: D conditions with similar phases and amplitudes (Figure 6B and C, Table S1), except for *Per2* in muscle, which peaked at approximately 2.6 h earlier in the stressed mice.

## Discussion

Chronic and social defeat types of stress induce depression and post-traumatic stress disorder (PTSD) with circadian rhythm abnormalities [23], [24]. These stressors are unsuitable for studies of circadian behavior because time cues are induced by exposing animals to stress once daily at a specific time. So far, an appropriate experimental system that can assess circadian locomotor activity during continuous exposure to a stressor has not been established. Here, we describe our novel system of continuous stress called PAWW. This type of stress induced hyperactivity during the early daytime, reduced locomotor activity during the nighttime, resulted in functional impairments while awake, and affected the quality and duration of sleep episodes. Our data were consistent with previous findings of activity patterns that correlate with sleep-wake behavior [25].

Table 1 compares our novel PAWW stress with conventional stressors. CMS based on continuous exposure to unpredictable stressors once a day for several weeks is an animal model of depression that affects locomotor activity in rats [23]. Exposure to CMS over a period of 6–8 weeks gradually reduces total and nighttime activity, but does not affect daytime activity. Single social defeat by placing a rat into the territory of a conspecific male rat for 1 h sharply suppresses nighttime locomotor activity [24]. The unique effect of PAWW stress on locomotor activity is an increase in daytime activity along with a decrease in nighttime activity. Furthermore, locomotor activity changed from the second day of exposure to PAWW stress. Rodents exposed to CMS and social defeat stress are not more active during the daytime [23], [24]. The circadian profile of BT was influenced and destabilized by PAWW stress, especially during the nighttime, whereas other types of stressors induce hyperthermia throughout the entire day. Exposure to PAWW stress obviously influenced both sleep rhythm and sleep architecture, whereas CMS and social defeat induce fragmented sleep [26], [27]. Thus, PAWW stress is unique in that it disrupts both locomotor activity and sleep rhythms. The novel PAWW stress takes advantage of the hedonistic properties of wheel running, which is a reward-like activity. However, hedonistic locomotor activity may cease to be pleasurable without appropriate rest.

**Table 1:** Comparison of PAWW stress with other conventional stress systems, chronic mild stress, social defeat stress and total sleep deprivation.

	PAWW stress	Chronic Mild Stress	Social Defeat stress	Total sleep deprivation
Stress Exposure duration and time	1 to >3 weeks	4–16 weeks	Once to several times	2 days
	Continuous	Once daily	1 h/day	Continuous
Circadian rhythm				
Measurement during stress Exposure	Yes	No	No	No
Total activity	Decrease	Decrease	Decrease	ND
Amplitude	Decrease	Decrease	Decrease	ND
Daytime activity	Increase	No	No	Increase
Body weight	Decrease	Not changed	Decrease	Decrease
Food intake	Increase	Decrease	Increase	Increase
Body temperature	Decreases in nighttime and unstable	Increases in daytime	Increased all day	Initial increase followed by large decrease
Corticosterone	Slightly increased in morning	Increased in daytime	Increased	Increased
Sleep	Sleep rhythm affected, Fragmented sleep, and reduced delta power	Fragmented sleep and increased REM sleep	Fragmented sleep and increased NREM sleep	Total deprivation
Release from stressor	Partially persists	Persists for several weeks	Persists for several weeks	Recovery within 1 day
References		22,27,32	26,33,37	34,36

ND: Not determined.

CMS generally serves as a model of PTSD and depression [11], [27], [28]. Total activity declined in a depression-like manner and the amplitude of activity was reduced in mice exposed to PAWW stress (Figure 1D). The recovery of locomotor activity from 1 week of PAWW stress varied intra- and inter-individually, but a partial effect of stress on locomotor activity persisted in 75% of the mice for 1 week after release from stress. The animals could be exposed to PAWW stress for over 3 weeks without adaptation. Psychiatric analysis of animals exposed to PAWW stress for longer periods will determine whether or not PAWW-stressed mice could serve as a model of human depression.

Mice in which circadian locomotor rhythms and sleep-wake cycles were blunted by continuous exposure to PAWW stress lost weight, despite increased food intake, and daytime plasma leptin levels were suppressed, indicating that the increased food intake was due to an enhanced sensation of hunger and destabilized BT. These results suggest that continuous PAWW stress negatively affects not only diurnal behavior but also energy balance, with which sleep homeostasis is closely associated [29]. The consequences of sleep loss caused by sleep deprivation or partial sleep restriction both in humans and rodents include decreased leptin levels and an increase in food intake and appetite [30]–[32].

Corticosterone levels became elevated on the first day of exposure to PAWW stress, but essentially fell to control levels on the 7^th^ day of exposure with similar diurnal oscillating profiles. However, these levels were slightly but significantly higher at the start of the rest period. CMS and total sleep deprivation elevated plasma corticosterone levels for longer periods than PAWW stress (from half of each day to all day) [33]–[35]. Plasma cortisol levels are remarkably elevated in patients with melancholia during the late evening and moderately elevated at other time points [36] and are moderately elevated in insomniacs during the late evening and early night, when most humans fall asleep and the levels of cortisol are low [37]. Our data are consistent with the features of human insomnia and depression. Catecholamines are also altered during stress in both mice and humans [36], [38]. Plasma catecholamines were elevated in the PAWW-stressed mice (Figure 3C and D). We speculate that the slightly higher corticosterone and catecholamine levels during the early morning are associated with the hyperactivation of locomotor activity at the normal time of sleep onset or that they are caused by the locomotor activity elicited by PAWW stress.

Circadian changes in BT are closely associated with sleep/wake timing, a reduction in BT at the beginning of the rest period during the smooth induction of sleep, and an increase in BT at the end of the rest period signals the time of awakening [39]. A moderate fall in core BT at the appropriate time might be necessary for good quality sleep; however, this did not occur in our stressed mice at the end of the dark period. Orexin-knockout mice have a narcolepsy-like phenotype with extreme sleep-wake fragmentation and dysregulated thermogenesis, but the core BT in these mice changes during the dark period, which coincides with repeated short sleep/wake cycles [40], and our model mice were similar to these. Disrupted thermogenesis might have contributed to the phenotype of the sleep-wake cycles of the mice under PAWW stress. The specific mechanisms underlying the effects of sleep loss on energy metabolism remain unknown; however, the PAWW stress model might facilitate studies of the molecular mechanisms underlying sleep regulation, stress responses, and systems that regulate energy metabolism.

Chronic continuous exposure to stress, from which sleep disorders are derived, is a hallmark of contemporary lifestyles. Insomnia is defined by time-dependent characteristics including difficulties in falling and/or staying asleep and poor quality sleep, followed by functional impairment while awake [41]. Animal models of stress-induced insomnia have been created using sleep deprivation techniques such as immobilization, electric shock, fear-conditioning, sensory stimulation, and maintenance on disks over water [14], [29], [42]. CMS and repeated social defeat can induce fragmented sleep in mice [12], [26]. However, these systems are invalid as models of sleep disorders with time-dependent characteristics. To date, compounds that can be used to treat insomnia have been assayed using healthy wild-type mice during the nighttime (active phase) [43]. Compared with these systems, the PAWW stress model will provide advantages for pharmacological studies of sleep disorders.

We speculate that PAWW stress did not affect the circadian clock in the SCN because the mice exhibited an obscure but substantive rhythmic locomotor activity under both L:D and D:D, and the time of locomotor activity onset after transfer from L:D to D:D corresponded to the timing predicted by the phase of their entrained rhythms (Figure 6). There was no significant difference in the free running periods during and after PAWW stress. Furthermore, PAWW stress did not affect the phases or amplitudes of circadian gene expression in the liver and muscle, which are synchronized by signals from the SCN. We believe that the peripheral clock system is not always affected by a change in the amplitude of activity, a hypothesis that the present findings support. Analyzing the effects of PAWW stress upon circadian core-component null mice will help to rule out the possibility of a downstream cascade of the canonical circadian machinery.

We have developed a novel stress exposure system, PAWW stress. Unlike other stress models, our PAWW stress model allowed wild-type mice to undergo stress for more than 3 weeks without complicated implementation or adaptation. Therefore, the PAWW stress model will be appropriate for analyzing the effects of treatment on stress-induced disorders of circadian rhythms and sleep, and for screening dietary supplements that could improve such conditions.

## Supporting Information

Figure S1 **The locomotor activities after release from PAWW stress.** Variable 24-h wheel-running activity in 4 mice under pre- (open circles, dashed line), PAWW (closed circles, solid line), and post- (shaded circles, gray line) stress conditions. The values represent averaged 1-h bin activity for 4 days per period and are plotted as means ± SEM. (A–C) Locomotor activity did not fully recover within 1 week after release from PAWW stress (RM two-way ANOVA, comparison between pre- and post-PAWW, F(1, 6) = 18.75 (A), 3.67 (B), and 35.10 (C), P<0.05). (D) Locomotor activity promptly returned to pre-stress levels (F(1,6) = 1.64, not significant).
(TIF)

Figure S2 **PAWW stress induced instabilities of core BT.** Representative diurnal BT profiles before and during PAWW stress (A). The white/black bars in each graph show the light and dark periods of the L:D cycle (ZT0, lights on; ZT12, lights off), respectively. The arrow indicates the time when the exposure to stress was started. BT that was previously stable became destabilized during PAWW stress. (B) Difference from averaged BT at ZT0–5 (L1), ZT6–11 (L2), ZT12–17 (D1), and ZT18–23 (D2). Deviations in BT at each time point (5-min bins) from the average temperature (for 1 h) were calculated and the mean value over 6 h was taken as the instability index of the BT. Continuous stress induced changeable BT in mice (RM-two way ANOVA, F(1,10) = 21.88, P = 0.001, the asterisk indicates a significant main effect).
(TIF)

Figure S3 **PAWW stress affected on a sleep architecture.** Duration of wakefulness, REM sleep, and NREM sleep under pre-stress, acute stress, and 7 days of PAWW stress (A). Compared with the duration under the pre-stress conditions, acute stress significantly changed the length of wakefulness, REM sleep, and NREM sleep (asterisks, Student's t-test, P<0.05), but not PAWW stress for 1 week. (B) Representative hypnograms of mice before and during PAWW stress. Short sleep-wake transitions were repeated throughout the daytime. Representative averaged EEG power density during nighttime NREM sleep of the pre-stressed (open circles) and PAWW stressed (closed circles) mice. [44]
(TIF)

Table S1 Cosinor method analysis of plasma corticosterone levels and gene expressions of ***Per2*** **and** ***Bmal1*** in peripheral tissues.
(XLS)
