# Supplementary figures and images for: Continuous Exposure to a Novel Stressor Based on Water Aversion Induces Abnormal Circadian Locomotor Rhythms and Sleep-Wake Cycles in Mice
Source: PLoS One. 2013 Jan 30;8(1):e55452. doi: 10.1371/journal.pone.0055452 (PMC3559439; doi:10.1371/journal.pone.0055452)

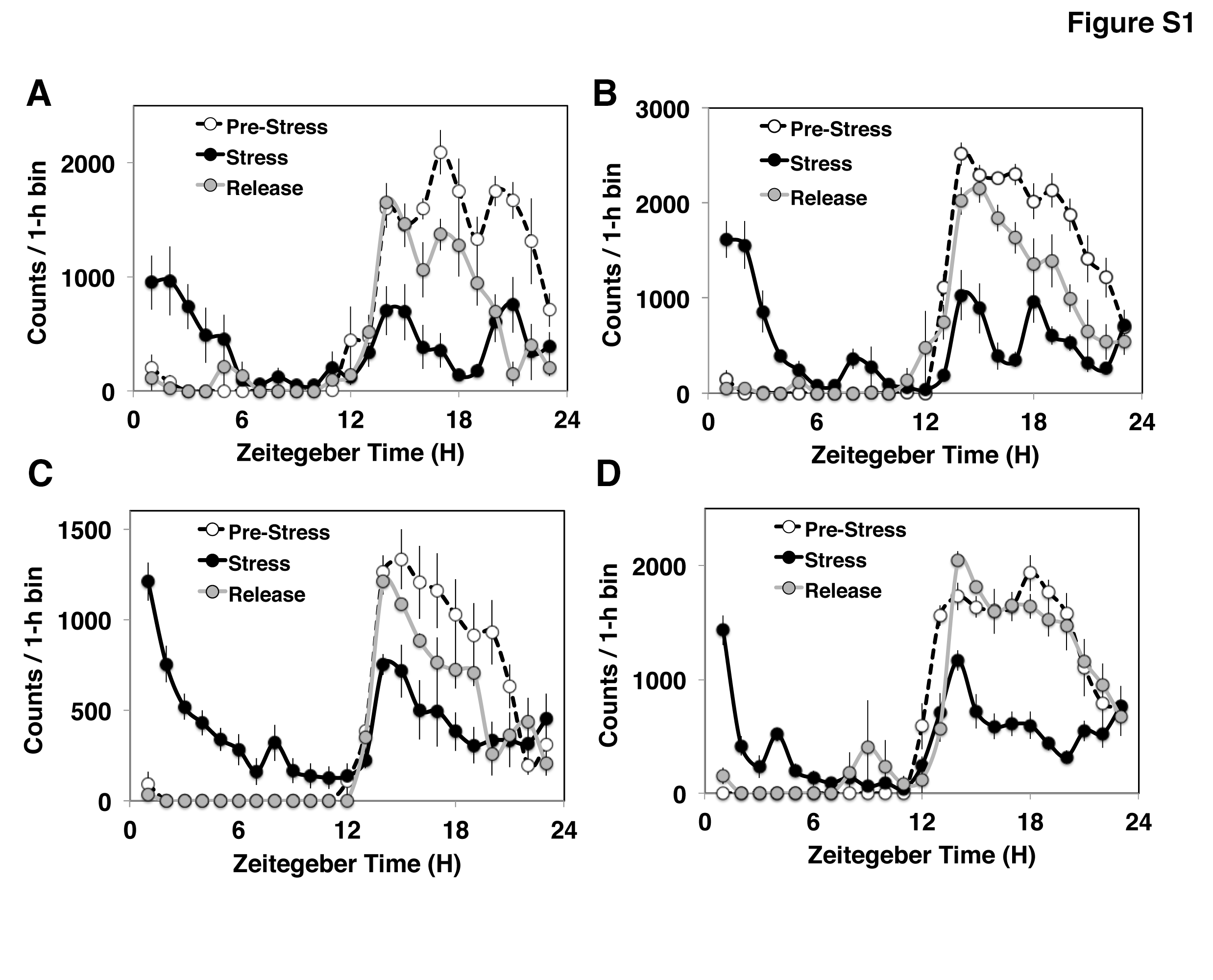

Supplement: Figure S1 — The locomotor activities after release from PAWW stress. Variable 24-h wheel-running activity in 4 mice under pre- (open circles, dashed line), PAWW (closed circles, solid line), and post- (shaded circles, gray line) stress conditions. The values represent averaged 1-h bin activity for 4 days per period and are plotted as means ± SEM. (A–C) Locomotor activity did not fully recover within 1 week after release from PAWW stress (RM two-way ANOVA, comparison between pre- and post-PAWW, F(1, 6) = 18.75 (A), 3.67 (B), and 35.10 (C), P<0.05). (D) Locomotor activity promptly returned to pre-stress levels (F(1,6) = 1.64, not significant). (TIF) [file pone.0055452.s001.tif]

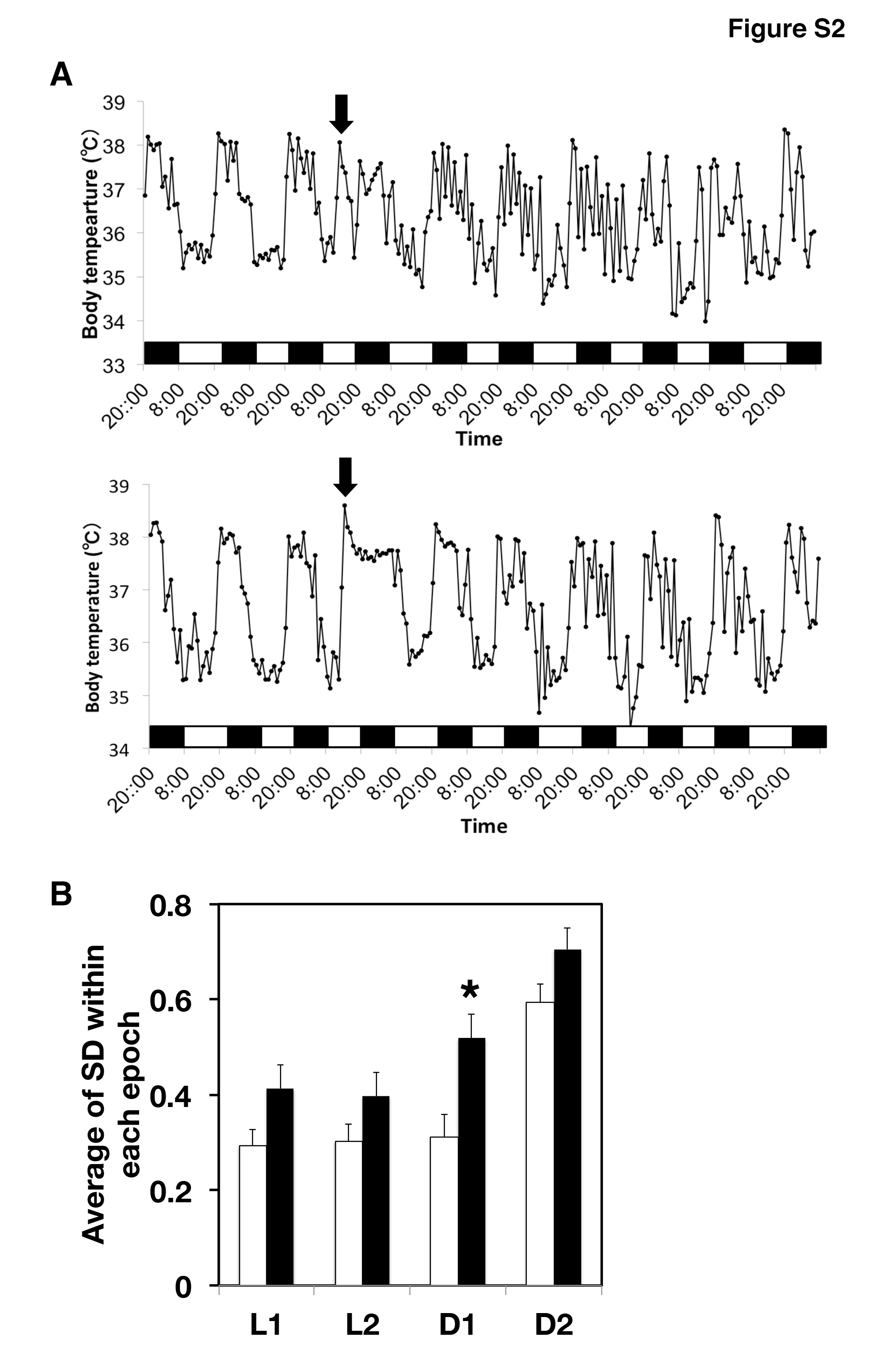

Supplement: Figure S2 — PAWW stress induced instabilities of core BT. Representative diurnal BT profiles before and during PAWW stress (A). The white/black bars in each graph show the light and dark periods of the L:D cycle (ZT0, lights on; ZT12, lights off), respectively. The arrow indicates the time when the exposure to stress was started. BT that was previously stable became destabilized during PAWW stress. (B) Difference from averaged BT at ZT0–5 (L1), ZT6–11 (L2), ZT12–17 (D1), and ZT18–23 (D2). Deviations in BT at each time point (5-min bins) from the average temperature (for 1 h) were calculated and the mean value over 6 h was taken as the instability index of the BT. Continuous stress induced changeable BT in mice (RM-two way ANOVA, F(1,10) = 21.88, P = 0.001, the asterisk indicates a significant main effect). (TIF) [file pone.0055452.s002.tif]

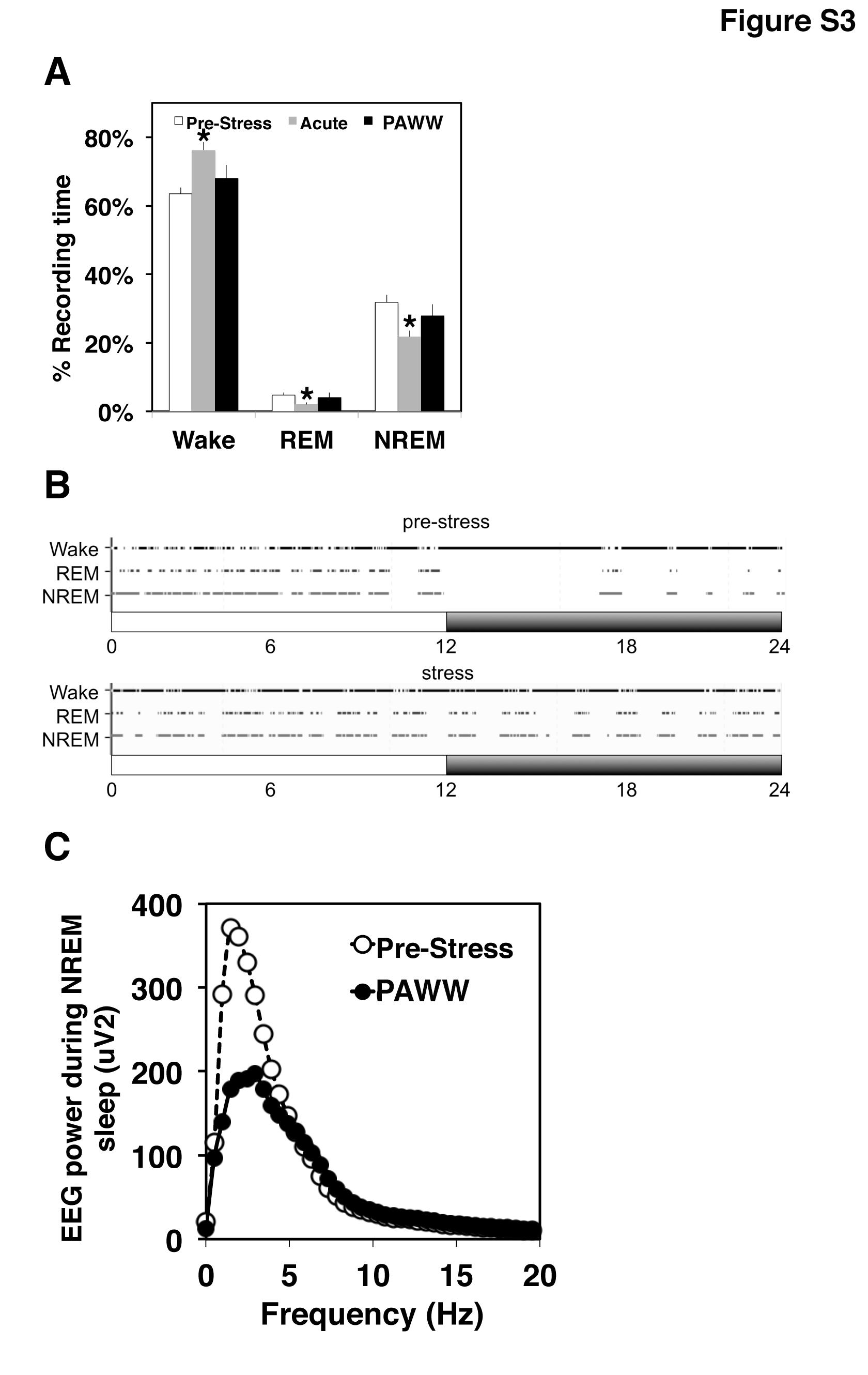

Supplement: Figure S3 — PAWW stress affected on a sleep architecture. Duration of wakefulness, REM sleep, and NREM sleep under pre-stress, acute stress, and 7 days of PAWW stress (A). Compared with the duration under the pre-stress conditions, acute stress significantly changed the length of wakefulness, REM sleep, and NREM sleep (asterisks, Student's t-test, P<0.05), but not PAWW stress for 1 week. (B) Representative hypnograms of mice before and during PAWW stress. Short sleep-wake transitions were repeated throughout the daytime. Representative averaged EEG power density during nighttime NREM sleep of the pre-stressed (open circles) and PAWW stressed (closed circles) mice. [44] (TIF) [file pone.0055452.s003.tif]
